# Tuning of Thermoelectric Properties of MoSe_2_ Thin Films Under Helium Ion Irradiation

**DOI:** 10.1186/s11671-022-03665-9

**Published:** 2022-02-10

**Authors:** Hyuk Jin Kim, Nguyen Van Quang, Thi Huong Nguyen, Sera Kim, Yangjin Lee, In Hak Lee, Sunglae Cho, Maeng-Je Seong, Kwanpyo Kim, Young Jun Chang

**Affiliations:** 1 Department of Physics, University of Seoul, 163 Siripdaero, Dongdaemun-gu, Bldg 14-217, Seoul, 02504 Republic of Korea; 2 Department of Physics and Energy Harvest Storage Research Center, University of Ulsan, Ulsan, 44610 Republic of Korea; 3 Department of Physics, Chung-Ang University, Seoul, 06974 Republic of Korea; 4 Department of Physics, Yonsei University, Seoul, 03722 Republic of Korea; 5 Center for Spintronics, Korea Institute of Science and Technology, Seoul, 02792 Republic of Korea; 6 Department of Smart Cities, University of Seoul, Seoul, 02504 Republic of Korea

**Keywords:** MoSe_2_, Helium ion irradiation, Thermoelectric property, Seebeck coefficient

## Abstract

Transition metal dichalcogenides have attracted renewed interest for use as thermoelectric materials owing to their tunable bandgap, moderate Seebeck coefficient, and low thermal conductivity. However, their thermoelectric parameters such as Seebeck coefficient, electrical conductivity, and thermal conductivity are interdependent, which is a drawback. Therefore, it is necessary to find a way to adjust one of these parameters without affecting the other parameters. In this study, we investigated the effect of helium ion irradiation on MoSe_2_ thin films with the objective of controlling the Seebeck coefficient and electrical conductivity. At the optimal irradiation dose of 10^15^ cm^−2^, we observed multiple enhancements of the power factor resulting from an increase in the electrical conductivity, with slight suppression of the Seebeck coefficient. Raman spectroscopy, X-ray diffraction, and transmission electron microscopy analyses revealed that irradiation-induced selenium vacancies played an important role in changing the thermoelectric properties of MoSe_2_ thin films. These results suggest that helium ion irradiation is a promising method to significantly improve the thermoelectric properties of two-dimensional transition metal dichalcogenides.

**Graphical Abstract:**

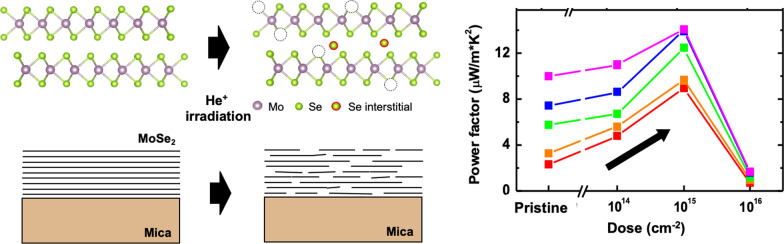
Effect of He^+^ irradiation on thermoelectric properties of MoSe_2_ thin films.

## Introduction

Transition metal dichalcogenides (TMDCs) have attracted considerable attention as two-dimensional (2D) material candidates beyond graphene owing to their unique electronic, optical, mechanical, chemical, and thermal properties [1]. They comprise one transition metal atom (i.e., Mo or W) and two chalcogen atoms (i.e., S, Se, or Te). The hexagonal metal layer is sandwiched by two hexagonal chalcogen layers, while the interface between the chalcogen layers is weakly bonded through van der Waals bonding. Such weak van der Waals bonding allows exfoliation of individual layers, stacking of arbitrary heterostructures, intercalation of charged ions (Li^+^ or Na^+^), and low thermal conductivity in the vertical direction [2–4]. In particular, semiconducting compounds, such as MX_2_ (M = Mo, W, or Re; X = S or Se), have been intensively investigated for their novel electronic and optical properties such as direct–indirect band gap transition [1, 5], large exciton signal [6], valley polarization [7], anisotropic electronic properties [8], and thermoelectric properties [9, 10].

Thermoelectric properties of TMDCs, especially, have been investigated because of diverse electronic properties, low thermal conductivity, and moderate Seebeck coefficient [11]. The energy conversion efficiency of thermoelectric materials is related to a dimensionless figure of merit *ZT* given by $ZT = \frac{{\alpha^{2} \sigma }}{\kappa }T$, where *α* is the Seebeck coefficient, *σ* is the electrical conductivity, *κ* is the thermal conductivity, *T* is the average temperature, and *α*^2^*σ* is the thermoelectric power factor. For the improvement of the thermoelectric performance, or achievement of a high *ZT* value, *α* and *σ* should be increased and *κ* should be decreased. However, these three parameters are adversely interdependent, and therefore, tuning one parameter inevitably changes the other parameters. Attempts have been made to overcome this problem, for instance, through the fabrication of nanostructures or ion-beam-induced defect engineering. Nanostructures, such as nanowires, nanosheets, nanohole patterns, or superlattices, have considerably low thermal conductivity owing to increased phonon scattering [12].

Irradiation of TMDCs with ion beams, such as hydrogen or helium ions (He^+^), induces defects, such as dislocations, interstitials, and vacancies, and thereby strongly influences the crystalline order, electrical, and thermoelectric properties [13–15]. He^+^ has a low mass, a small atomic radius, and a low scattering cross section in comparison with heavy atoms, such as Ne^+^, Ar^+^, and Kr^+^. The heavier atoms often ablate the sample surface and form amorphous phase, so that they are employed for surface removal or sputtering. On the other hand, He^+^ can be employed for wide range applications, such as gentle modification of whole thin film devices [15], helium ion microscopy [16], and focused ion milling [17], depending on the acceleration energy and dose. The Seebeck coefficient is obtained from the Mott relation $\alpha \propto m^{*} /n$, where m^*^ is the effective mass and *n* is the carrier concentration [11], and the electrical conductivity can be written as $\sigma = en\mu$, where *e* is the carrier charge and *µ* is the electrical mobility. Ion irradiation increases the carrier concentration but also induces structural defects, which then decreases the carrier mobility and the lattice thermal conductivity. Such intertwined influences of ion irradiation on the carrier transport properties may result in nonlinear behaviors of thermoelectric properties for the ion irradiation. In addition, the suppressed lattice thermal conductivity may enhance the ZT value.

Recent theoretical calculations have yielded a high *ZT* (= 1) for semiconducting TMDCs, that is, Mo(S, Se)_2_ and WSe_2_ [18–20]. Based on the first-principles calculations or molecular dynamics method, the intrinsic electrical and thermal properties of pristine TMDCs are theoretically studied and their differences with the experiments are maybe due to the pristine nature of their simulation models. Notably, a high Seebeck coefficient (*α* = 10 mV/K) [9] and large variations of thermal conductivities (0.05–40 W/(m K)) [4, 21, 22] have been experimentally observed. Furthermore, the mechanical flexibility of TMDCs and their atomically thin layers render them promising materials for use in wearable electronics; it has been shown that a prototype thermoelectric wearable device with a combination of chemically exfoliated *n*-type WS_2_ and *p*-type NbSe_2_ generates an electrical power when a temperature gradient exists on the human body [23]. However, in-depth studies on the optimization of thermoelectric properties of TMDCs thin films via ion irradiation are scarce.

In this study, we investigated the use of He^+^ irradiation for improving the thermoelectric properties of MoSe_2_ thin films. Raman spectroscopy and high-resolution X-ray diffraction (HRXRD) measurements were made to analyze changes in the phonon vibration mode and crystal structure of the thin films, respectively, and transmission electron microscopy (TEM) and energy-dispersive X-ray spectroscopy (EDS) were used to detect microscopic atomic disorders and determine chemical stoichiometries, respectively. Furthermore, the dependence of the Seebeck coefficient and electrical conductivity on the irradiation dose was examined.

## Methods

MoSe_2_ thin films were grown on a mica muscovite substrate in a high-vacuum co-evaporation chamber with a base pressure below 5 × 10^–8^ Torr. High-purity Mo (99.95%) and Se (99.999%) were simultaneously evaporated from an electron-beam evaporator and a Knudsen cell at rates of 0.1 and 1.5 Å/s, respectively, with the substrate temperature being maintained at 260℃ [24, 25]. The film thicknesses were determined through calibration with a quartz crystal oscillator and verified by cross-sectional TEM analysis, and they were about 30 nm. Thin film growth was followed by cooling, without post-annealing. The thin films were irradiated with a 30 keV He^+^ beam obtained from an ion accelerator installed at the Korea Multi-purpose Accelerator Complex (KOMAC, Gyeongju, Korea). The selected He^+^ doses were 10^14^, 10^15^, and 10^16^ cm^−2^, and they are hereafter referred to as He-14, He-15, and He-16, respectively. After the He^+^ irradiation, the samples were taken from the facility after a few days because the radioactive level of the samples should be below the certain safety level. The previous real-time monitoring of electrical properties of graphene during irradiation shows fast saturating behavior just after the irradiation event [26, 27]. Therefore, we may expect the most physical properties of irradiated samples are saturated to certain values when we analyze afterward.

Raman spectroscopy measurements were taken using a 532-nm excitation laser source with a fixed power (30 mW) and a fixed acquisition time (60 s) at room temperature. Scattered light from the samples was analyzed using a single-grating monochromator with a focal length of 50 cm, and it was detected by a liquid–nitrogen-cooled charge-coupled-device detector [28]. HRXRD measurements were made using an in-house X-ray diffractometer (D8, Bruker) with Cu Kα_1_ radiation, and the film microstructure was studied using TEM (JEM-2100F and JEM-ARM200F, JEOL) at an accelerating voltage of 200 kV. Cross-sectional film samples for TEM were fabricated by focused-ion-beam (Helios Nanolab 450, FEI), and plan-view TEM samples of MoSe_2_ film were prepared by the wet transfer method. Chemical stoichiometries were analyzed using EDS from field-emission scanning electron microscopy (FE-SEM, SU8101, Hitachi) measurements. The Seebeck coefficient and electrical conductivity were simultaneously measured from 300 to 420 K by using a home-built thermoelectric measurement system, which is described elsewhere [29].

## Results and Discussion

**Fig. 1:**
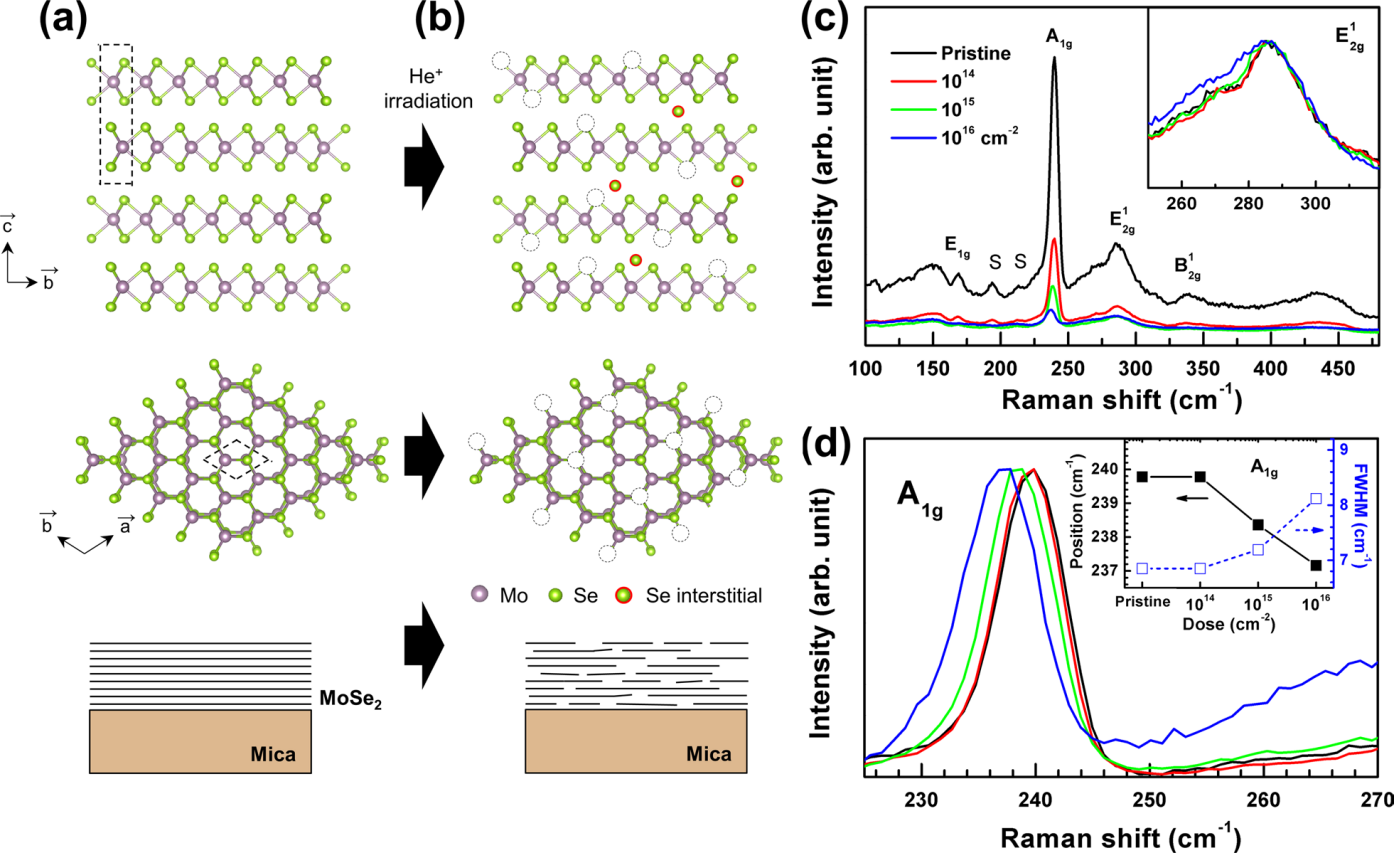
Crystal structures of 2HMoSe_2_ and schematics of MoSe_2_ film samples **a** before and **b** after He^+^ irradiation. The black dashed boxes indicate unit cells of MoSe_2_. **c** Raman spectra of the unirradiated MoSe_2_ film (black curve) and MoSe_2_ films irradiated with different He^+^ doses: 10^14^ (red), 10^15^ (green), and 10^16^ cm^−2^(blue). Peaks attributed to the mica substrate are labeled “S.” The inset shows that the normalized E^1^_2g_ peaks did not shift. **d** Normalized A_1g_ peaks showing gradual red shift with the variation of the ion dose. The inset shows the position and FWHM of the A_1g_ peaks as a function of the ion dose

Figure 1a shows an atomic model of 2H phase MoSe_2_; top and side views of the compound are shown, and the dashed boxes indicate unit cells. MoSe_2_ has a trigonal prismatic crystal structure, D_3h_ point group symmetry, and a 2D layered structure with van der Waals bonding [30]. We chose mica substrates for fabricating flat films and minimized wrinkle formation during their growth [24]. Mica substrates have been widely used for van der Waals epitaxial growth of TMDC thin films owing to their atomically flat surface and absence of dangling bonds [31]. Figure 1c shows Raman spectra of He^+^-irradiated MoSe_2_ thin films. The pristine sample shows A_1g_, E_1g_, E^1^_2g_, and B^1^_2g_ modes, which correspond to out-of-plane (A_1g_ and B^1^_2g_) and in-plane (E_1g_ and E^1^_2g_) phonon vibrations. The peak positions are consistent with those of MoSe_2_ bulk and thick films [5]. The mica substrate contributed three small peaks, labeled by the letter “S.” Raman peak intensities are inversely proportional to defect formation and disorder for a crystalline lattice. As the He^+^ dose increased, the Raman peak intensities reduced in the entire measurement range, indicating that the He^+^ beam introduced significant defects or disorder in both the films and substrates. Notably, the A_1g_ peak shifted from 239.8 cm^−1^ (pristine) to 237.2 cm^−1^ (He-16) and its full width at half-maximum (FWHM) also increased from 6.9 to 8.1 cm^−1^ (Fig. 1d) as the He^+^ dose increased. By contrast, the inset of Fig. 1c shows that the E^1^_2g_ peak did not shift. Raman peak positions of TMDCs are sensitive to the layer thickness [5], presence of the thermal effect [32], number of point defects (i.e., vacancies) [33], and strain state [34], and among these, a change in the number of point defects or strain state would be the most probable cause for the change in the A_1g_ peak. Furthermore, notably, only the out-of-plane vibration A_1g_ mode is sensitively shifted, which rules out the possibility of any changes in the strain state. Thus, Raman analysis indicated the generation of a significant amount of defects and disorder in the MoSe_2_ thin films by He^+^ irradiation, which is consistent with the case of He^+^-irradiated MoS_2_ [13].

**Fig. 2:**
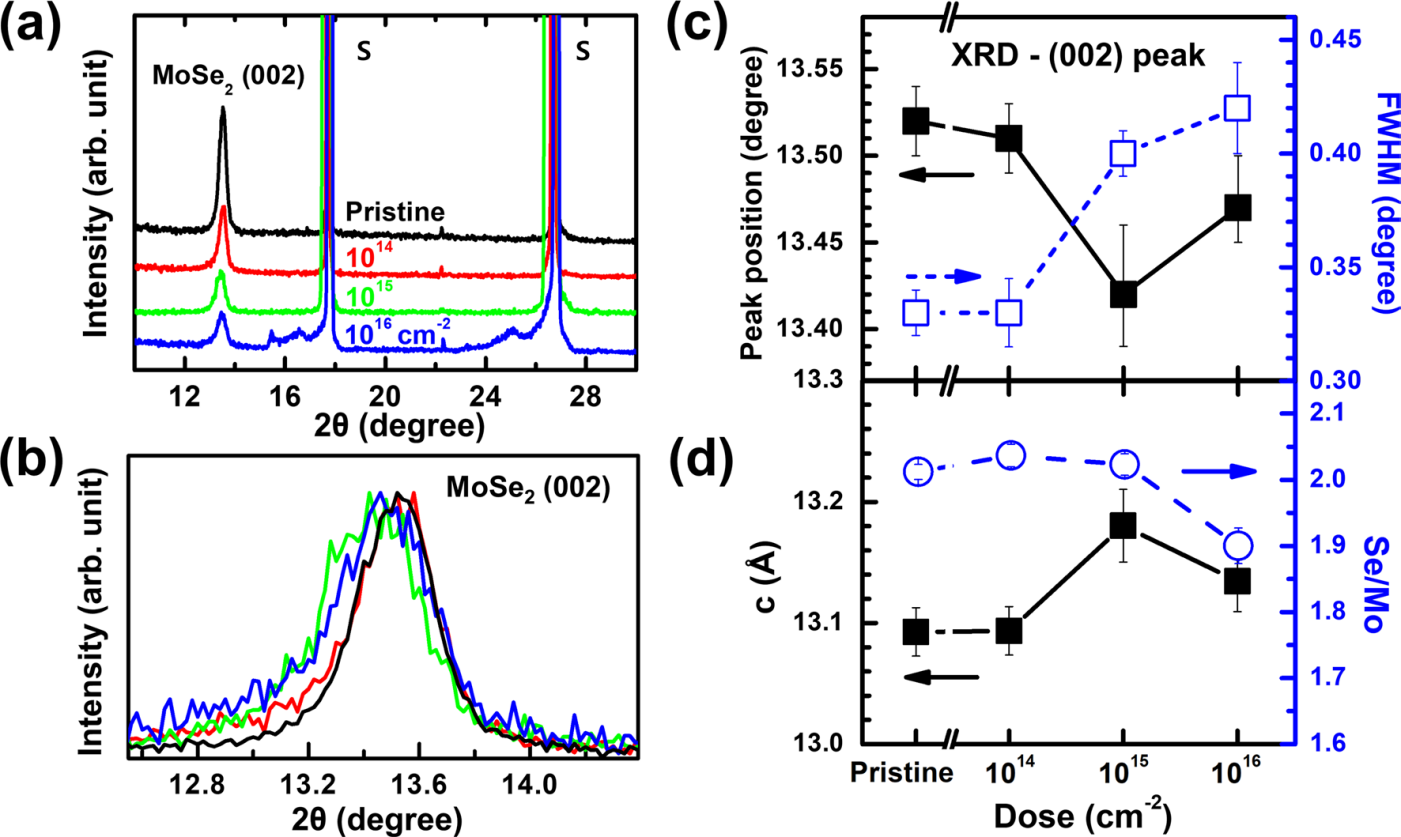
**a** HRXRD patterns of He^+^-irradiated MoSe_2_ films on mica substrates (“S” denotes mica substrate peaks) and **b** a magnified view of the MoSe_2_ (002) peaks with a distinctive shift. **c** Peak positions and FWHM of MoSe_2_ (002) as a function of the irradiation dose. **d** The *c*-axis lattice parameter and Se/Mo ratio obtained from HRXRD and EDS, respectively, as a function of the irradiation dose

To compare the crystalline state for different irradiation doses, we performed HRXRD measurements, shown in Fig. 2a, for the series of MoSe_2_ thin films. The pristine MoSe_2_ film showed a characteristic (002) peak at 13.5°, which matched that of the 2H MoSe_2_ phase. The *c*-axis lattice constant was determined to be 13.09 Å, which was close to those of bulk and thin films [25, 30]. Absence of peaks other than the strong (002) peak indicated well-stacked layered growth along the *c*-axis of the mica substrates. The strong peaks at 2θ = 17.5° and 26.5° corresponded to the (001) and (002) planes of the mica substrate, respectively [35]. When the films were irradiated with the He^+^ beam, the (002) peak’s intensity gradually decreased and shifted toward lower angles. As evident in Fig. 2b, the normalized MoSe_2_ (002) peak shifted and its FWHM increased. We compared the peak positions and FWHM as a function of the irradiation dose, as shown in Fig. 2c. Interestingly, both peak position and FWHM values started to change significantly when the He^+^ dose exceeded 10^15^ cm^−2^. Consequently, the extracted *c*-axis lattice parameter increased by 0.4% (Fig. 2d), consistent with the shift in the A_1g_ peak (out-of-plane mode).

To examine stoichiometric variation, we performed EDS analysis from FE-SEM measurements without sample transfer. Atomic percentage ratios of Se/Mo were extracted from the EDS spectrum for all the samples, and they are shown in Fig. 2d. The Se/Mo ratio ranged from 2.01 (pristine sample) to 1.90 (He-16 sample). We speculate that the initial He^+^ irradiation (He-14) introduced crystalline disorder without affecting the Se/Mo ratio, possibly generating atomic vacancies and interstitials. A much higher irradiation dose (He-16) reduced the Se content and significantly disturbed the in-plane crystalline order, as shown schematically in Fig. 1b. First-principles studies have shown that the formation of chalcogen vacancies is energetically preferred over transition metal vacancies [36]. The mass of Se is 1.2 times smaller than that of Mo, and consequently He^+^ ions transfer more energy to Se atoms than to Mo atoms during collisions. Notably, preferential sputtering of light atoms in MoS_2_ has been observed for irradiation with He^+^ and electron beams [13].

**Fig. 3:**
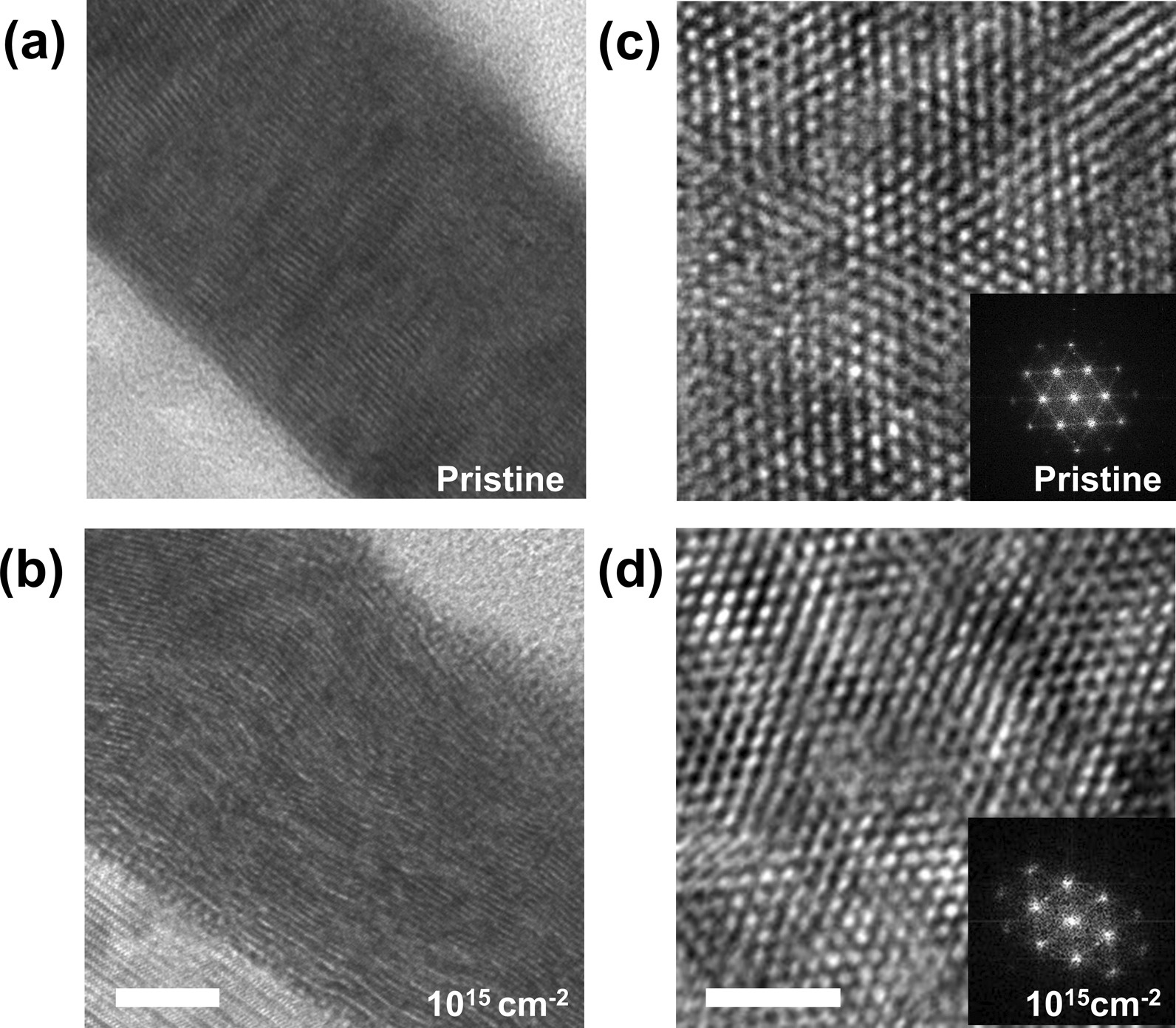
Cross-sectional TEM images of **a** the pristine sample and **b** the He-15 sample (scale bar = 10 nm). Plan-view TEM images of **c** the pristine sample and **d** the He-15 sample (scale bar = 2 nm). The insets show the corresponding FFT images

To understand the microscopic effects of He^+^ irradiation of layered MoSe_2_, we performed a series of TEM analyses, and TEM images of the pristine sample and He-15 sample are shown in Fig. 3. Cross-sectional TEM was used to study the layered structure, and plan-view TEM was used to investigate the lattice arrangement of atoms. In Fig. 3a, the low-magnification bright-field cross-sectional TEM image shows uniform film growth with a thickness of 30 nm. The pristine sample shows a well-stacked layered structure with uniform contrast and thickness on the surface plane of the mica substrate. This indicates that atomically flat mica substrates are ideal for fabricating layered MoSe_2_ thin films [31]. On the other hand, the He-15 sample shows a more disordered stacking with largely wiggling patterns, indicating the presence of grain boundaries and dislocations, as expected from the Raman and HRXRD analyses (Fig. 3b). Figure 3c, d shows plan-view TEM images with their fast Fourier transformation (FFT) images. The pristine sample showed a well-ordered hexagonal lattice, and the lattice constant (*a* = 3.3 Å) determined from the plan-view TEM images corresponded to that of bulk 2H MoSe_2_ [30]. Plan-view TEM images also show local structural disorders, such as grain boundaries, defects, and dislocations. Although some of defects may have originated during the transfer of the fabricated film onto the TEM grid, we examine the possibility of film damage after He^+^ irradiation. The FFT image of the pristine sample showed quite sharp diffraction peaks (inset of Fig. 3c). In the He-15 sample, the degree of disorder significantly increased, while the corresponding FFT showed broadened patterns. TEM analysis provided microscopic evidence of crystalline disorders or defects for He^+^ dose beyond a threshold value. When increasing the He^+^ dose, both the Raman and XRD peaks are drastically suppressed, and the TEM images demonstrate local atomic structural disorders. Therefore, through the comprehensive analyses, such as Raman, XRD, and TEM, we demonstrate that crystalline disorder increases as the He^+^ dose increases.

**Fig. 4:**
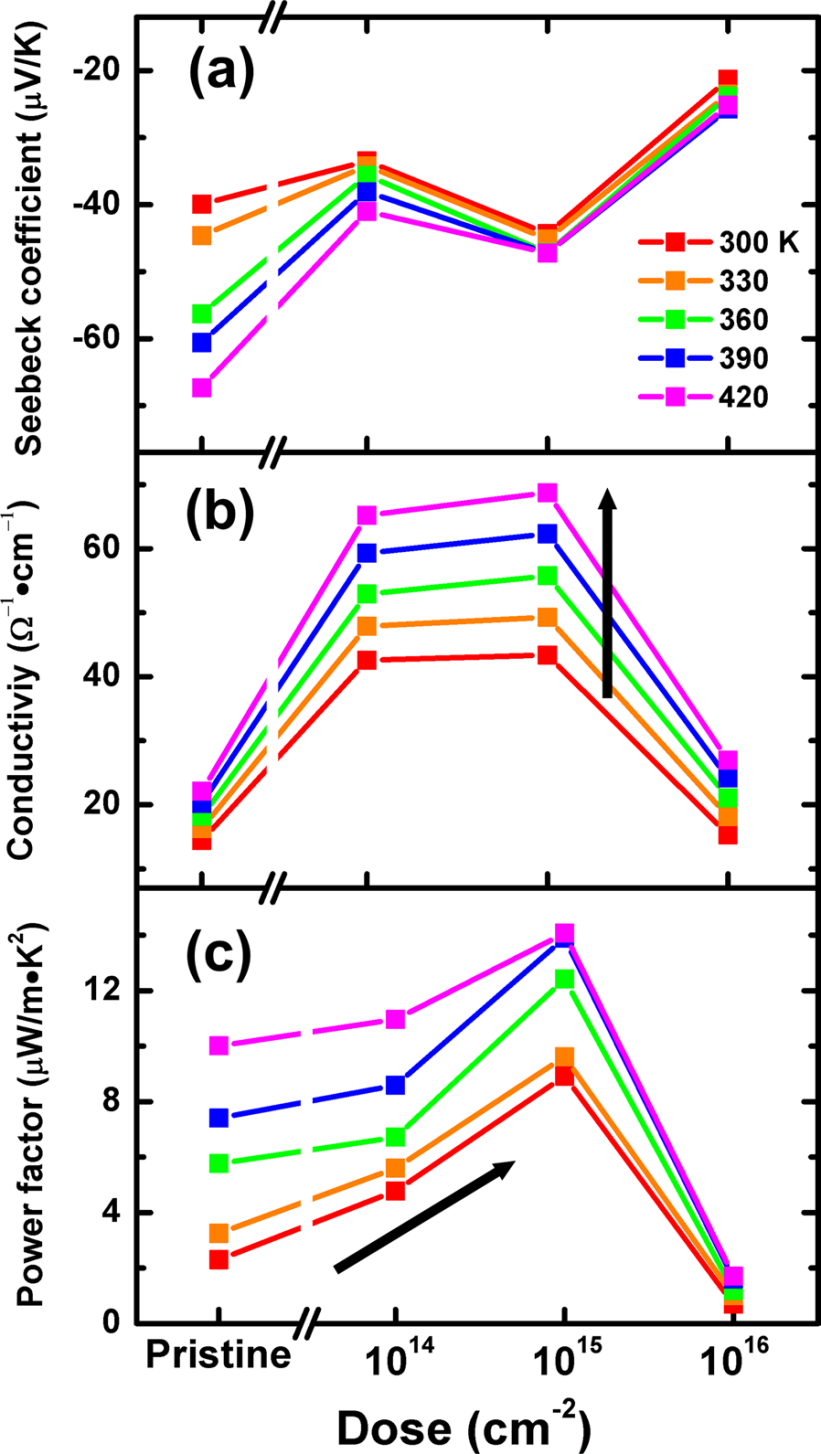
Thermoelectric properties of the He^+^-irradiated MoSe_2_ films: **a** Seebeck coefficient, **b** electrical conductivity, and **c** power factor of the He^+^-irradiated thin films as a function of the ion dose in the temperature range 300–420 K

We next performed the thermoelectric characterization of the films. The uniformly continuous nature of the films and the insulating mica substrate enabled us to perform temperature-dependent Seebeck and electrical conductivity measurements of the as-grown films from 300 to 420 K. In Fig. 4a, all samples show negative Seebeck coefficients, indicating that electrons were the dominant carriers. The pristine sample shows strong temperature dependence of the Seebeck coefficient. The temperature dependence is considerably reduced with an increase in the irradiation dose, while the magnitude of the Seebeck coefficient gradually decreases. Figure 4b shows the electrical conductivity as a function of the temperature and dose. All the samples show similar insulating behavior, with the conductivity increasing with the temperature (the arrow in Fig. 4b). With an increase in the ion dose, the conductivity sharply increased up to 10^15^ cm^−2^ and then dropped at 10^16^ cm^−2^. The thermoelectric power factor showed nonlinear dependence on the dose (Fig. 4c), and it showed a gradual increase when the dose was increased up to 10^15^ cm^−2^. However, the sharp decrease in both Seebeck coefficient and conductivity minimized the thermoelectric power factor. Notably, the maximum power factor value of 13 μW/m K^2^ was obtained at 420 K for the He-15 sample.

We explain the observed thermoelectric properties based on our microscopic understanding of the MoSe_2_ films under He^+^ irradiation. From the analysis discussed above, we note that He^+^ irradiation initially induced crystalline defects and then degraded the lattice structure, apart from introducing a significant number of Se vacancies. A small number of defects, such as vacancies or interstitials, are likely to increase the carrier concentration. An increase in the number of carriers enhances the conductivity but decreases the Seebeck coefficient, in accordance with the Mott relation, as discussed in Introduction section. However, a higher dose of ion irradiation (He-16) significantly damaged the crystalline phase by introducing Se vacancies. The resulting high level of structural disorder caused significant scattering of charge carriers and degraded the carrier mobility, which explains degradation of the conductivity. In other words, the dose increases the carrier concentration but also introduces structural disorders, which then diminishes the carrier mobility. Such intertwined effects of ion dose on the carrier transport characteristics explain the nonlinear tendency of thermoelectric properties with respect to the He^+^ dose. It is also noted that the structural disorder also induced scattering of phonon transport and suppress the lattice thermal conductivity, which then may increase the ZT value of the materials [11].

Finally, our result demonstrates that the use of ion irradiation is a very powerful approach to enhance the thermoelectric performance of 2D materials. The maximum power factor value of 13 μW/m•K^2^ was obtained at 420 K, which is comparable to other TMDC nanosheets, single-walled nanotubes, and some organic materials [23, 37, 38]. We also observed that optimum irradiation (10^15^ cm^−2^) could increase the power factor value of the MoSe_2_ films by ~ 4 times at 300 K. In view of the reported power factor values of TMDC materials (5–80 μW/m K^2^) [9–11], it would be of considerable interest to use the ion irradiation method to improve their thermoelectric properties. Also, the similar approach of He^+^ irradiation to improve thermoelectric performance can be extended to improve thermoelectric device performance by inserting a He^+^ irradiation step into device fabrication process. In a previous report, flexible thermoelectric device applications utilized similar TMDC compounds, such as WS_2_-NbSe_2_, in which power factor of WS_2_ component reaches 6 μW/m•K^2^ [23]. Furthermore, this approach is well matched in typical thermoelectric materials such as Bi_2_Te_3_ and Sb_2_Te_3_. Although Bi_2_Te_3_ is differentiated from TMDC in that Bi_2_Te_3_ has rhombohedral crystal structure and 3D topological insulating electronic structure, ion irradiation improves thermoelectric characteristics by a factor of two in Bi_2_Te_3_ nanorod and organic/Bi_2_Te_3_ composite film [39]. Therefore, the effectiveness of the ion irradiation technique suggests that it can be used for universal tuning of the thermoelectric properties of various materials, including TMDCs and other 2D layered materials.

## Conclusion

To summarize, we investigated the use of He^+^ irradiation for controlling the thermoelectric properties of MoSe_2_ thin film by varying the irradiation dose. The use of an irradiation dose beyond a threshold value caused crystalline defects and chemical disorder, which were evidenced by Raman, HRXRD, and TEM analyses. We found that irradiation-induced defects increased the electrical conductivity while slightly reducing the Seebeck coefficient. The irradiation dose was varied up to 10^16^ cm^−2^, and the maximum value of the power factor of the MoSe_2_ thin films was obtained at 10^15^ cm^−2^. Along with the low thermal conductivity values of TMDCs, the observed increase in the power factor with the irradiation dose indicated high thermoelectric performances of the TMDCs. Furthermore, our result shows that the use of helium ion irradiation and irradiation-based approaches has high potential for use in the development of high-performance thermoelectric devices.

## Data Availability

The datasets supporting the conclusions of this article are included within the article.

## Abbreviations

TMDC: Transition metal dichalcogenide
He^+^: Helium ion
HRXRD: High-resolution X-ray diffraction
TEM: Transmission electron microscopy
EDS: Energy-dispersive X-ray spectroscopy
FE-SEM: Field-emission scanning electron microscopy
FWHM: Full-width half maximum
FFT: Fast Fourier transformation
